# Oclusão do Apêndice Atrial Esquerdo e Implante de Sistema Bicaval: Relato do Primeiro Procedimento Transcateter Simultâneo em Paciente Idoso Grave

**DOI:** 10.36660/abc.20230454

**Published:** 2023-12-07

**Authors:** Marcos Cherem, Carlos Eduardo Bernini, Jamil Abdalla Saad, Dirceu Barbosa Dias, Marcio Sérgio Carvalho Silva, Ruthnea Aparecida Lazaro Muzzi

**Affiliations:** 1 Departamento de Cardiologia, Hemodinâmica e Cirurgia Cardiovascular Hospital Vaz Monteiro Lavras MG Brasil Departamento de Cardiologia, Hemodinâmica e Cirurgia Cardiovascular , Hospital Vaz Monteiro , Lavras , MG – Brasil; 2 Departamento de Hemodinâmica Hospital Felício Rocho Belo Horizonte MG Brasil Departamento de Hemodinâmica , Hospital Felício Rocho , Belo Horizonte , MG – Brasil; 3 Universidade Federal de Lavras Lavras MG Brasil Universidade Federal de Lavras , Lavras , MG – Brasil

**Keywords:** Apêndice Atrial, Veias Cavas, Cateterismo Cardíaco/métodos, Fibrilação Atrial, Insuficiência da Valva Tricúspide

A fibrilação atrial (FA) é uma arritmia frequente que está associada a um risco aumentado de morte, acidente vascular cerebral (AVC) e embolia periférica. ^1^ O fechamento do apêndice atrial esquerdo (AAE) como estratégia de profilaxia de eventos tromboembólicos em pacientes com FA utilizando oclusores percutâneos é uma opção de tratamento minimamente invasivo, especialmente em indivíduos com contraindicação à anticoagulação e cirurgia aberta. ^2^

O manejo da regurgitação tricúspide (RT) sintomática grave permanece sendo um grande desafio. ^3^ Neste contexto, o uso de dispositivos transcateter, como o implante de válvula cava heterotópica para aliviar a congestão venosa central, surgiu como uma estratégia para tratar indiretamente os efeitos sistêmicos da RT grave, torrencial. ^4^

Será relatado o primeiro caso de uma dupla intervenção percutânea torácica, de forma concomitante, visando ao mesmo tempo a oclusão do AAE e o implante do sistema TricValve em um paciente idoso e portador de quadro cardíaco grave.

## Relato de Caso

Paciente do sexo masculino, 87 anos, apresentando RT grave torrencial com importante repercussão hemodinâmica e em quadro de insuficiência cardíaca direita, episódios de tromboembolismo pulmonar e trombose venosa profunda de membros inferiores em acompanhamento no serviço de cardiologia do hospital desde 1996.

Paciente com graves comorbidades, como FA permanente em uso de enoxaparina profilática e impossibilidade de anticoagulação oral segura devido à insuficiência renal crônica. Além disso, apresentou concomitantemente hipertensão essencial estágio 3, hipertrofia ventricular esquerda, hipercolesterolemia e insuficiência venosa de membros inferiores. Mesmo em uso de metildopa, amiodarona, bisoprolol, enoxaparina, epoetina, dapagliflozina, furosemida, espironolactona, propatilnitrato, atorvastatina, alopurinol, evoluiu progressivamente com insuficiência tricúspide grave, dilatação de câmaras cardíacas direitas, congestão em veias cavas e átrio direito (AD), refratárias ao tratamento clínico otimizado, com progressiva piora da função renal. No ecocardiograma transtorácico observou-se hipertrofia concêntrica grave e desadaptada do ventrículo esquerdo, com comprometimento sistólico do ventrículo direito (VD), insuficiência tricúspide de grau importante, aumento biatrial importante, e moderada hipertensão pulmonar.

Em virtude do risco inaceitável para cirurgia cardíaca a céu aberto, foi indicado o tratamento percutâneo duplo, que consistiu no fechamento do AAE e colocação de válvula heterotópica nas cavas (TricValve®).

**Figura 1:**
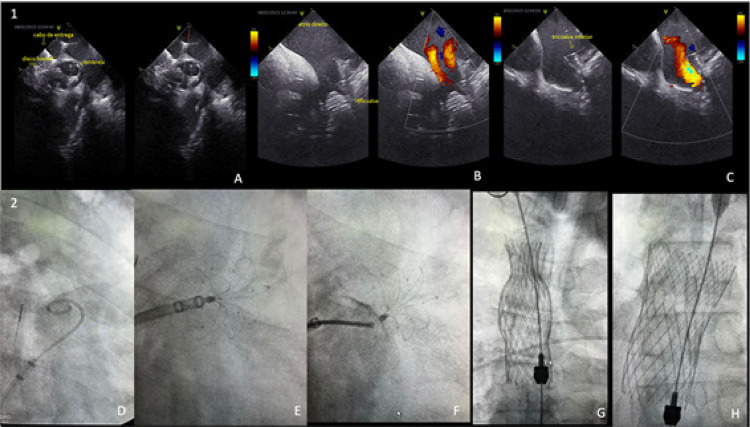
– 1: Imagens ecocardiográficas intracardíacas demonstrando em A) Oclusor do AAE, com o disco posicionado; B) Dispositivo TricValve na VCS; C) Dispositivo TricValve posicionado na VCI. 2: Imagens fluoroscópicas demonstrando em D) Injeção do contraste pelo cateter pigtail no AAE – tipo chicken wings; E) Liberação do dispositivo dentro do AAE; F) Dispositivo LAmbre liberado; G) Liberação do primeiro dispositivo de TricValve na VCS; H) Liberação do segundo dispositivo na VCI. AAE: apêndice atrial esquerdo; VCI: veia cava inferior; VCS: veia cava superior.

O ecocardiograma transesofágico foi realizado e evidenciou AAE apresentando anatomia tipo “ *chicken wing* ” bilobulada, sem trombos. O procedimento foi realizado sob sedação e inicialmente foram inseridos quatro introdutores venosos: um 5F e um 8F na veia femoral direita, um 5F na veia femoral esquerda e um 8F na veia jugular interna direita. Em seguida, foi realizada a punção transeptal para acessar o átrio esquerdo, com agulha de punção transeptal. Após, foi realizado a ecocardiografia intravascular (ICE) e a cateterização do AAE por angiografia verificando as medidas prévias. Prosseguiu-se com a colocação da bainha de entrega no AAE (técnica de Seldinger). Em seguida, foi implantado dispositivo LAmbre® 3236, com *oversizing* de 24% para uma implantação mais profunda no AAE, apoiado no lóbulo superior. O dispositivo foi liberado com a “ *umbrella* ” profunda, disco bem-posicionado no orifício e sem shunt residual (Figura 1: 1A, 2D-F).

Em seguida, o cateter ICE foi reposicionado no AD para visualização da veia cava superior. Introduziu-se fio-guia Lunderquist na veia subclávia direita e um cateter *pigtail* foi inserido pelo introdutor da veia femoral esquerda na artéria pulmonar direita. Com auxílio do ICE e *pigtail* , a válvula da veia cava superior (TricValve® 29 mm) foi posicionada e liberada, garantindo o bulbo do dispositivo na posição adequada, entre a veia inominada e a junção veno-atrial. Após a liberação, o ICE confirmou a ausência de shunt residual (Figura 1: 1B e 2G).

Posteriormente, o cateter ICE foi reposicionado no introdutor da veia jugular interna direita e avançado até o AD. Com visualização adequada da junção da veia cava inferior com o AD e do cateter *pigtail* na artéria pulmonar direita, foi realizada a colocação da válvula da veia cava inferior (TricValve 39 mm), garantindo fluxo adequado nas veias supra-hepáticas sem vazamento peri-protético (Figuras1: 1C e 2H). Após a liberação da válvula, foram retirados o Lunderquist e introdutores. O paciente teve alta hospitalar utilizando as mesmas medicações anteriores e apresenta quadro estabilizado, desde então sem internações por descompensação.

## Discussão

Esse relato descreve um caso de sucesso de colocação de implantes simultâneos em paciente idoso, com doença cardíaca grave e comorbidades que contraindicavam a cirurgia convencional por elevado risco de complicações e óbito.

O paciente do relato possuía RT como doença de base e quadro de FA permanente. Para reduzir as chances de evento embólico, optou-se pela oclusão do AAE com o dispositivo LAmbre. A oclusão transcateter do AAE tem se tornado cada vez mais popular como alternativa à anticoagulação para profilaxia de eventos tromboembólicos em pacientes com FA. ^1 , 5^ Em um estudo conduzido em 60 pacientes submetidos à oclusão percutânea do AAE, ^6^ foi discutida a possibilidade de vazamento peri-prótese. No paciente do relato isso não ocorreu, nem a formação de trombos ou evento cerebrovascular, demonstrando a eficácia e segurança terapêutica do uso de oclusores, especialmente em pacientes com FA e risco elevado de AVC. Contudo, é salientado ^7^ que, como ocorre com todo procedimento intervencionista, a curva de aprendizado desempenha papel essencial na oclusão percutânea do AAE, devendo ser feita por operadores experientes com ótimas habilidades, em colaboração com a equipe de cardiologia, como observado neste relato de caso, composta por experimentada equipe multidisciplinar.

Devido à disfunção sistólica do VD e regurgitação tricúspide grave, com repercussões sistêmicas, optou-se pelo implante do sistema TricValve. Foi descrito em estudo ^4^ que o sistema bicaval heterotópico surgiu como possível estratégia transcateter para tratar indiretamente os efeitos sistêmicos da RT grave, com características que diminuem sobremaneira o risco de embolização do implante. Não requer anestesia geral e não tem contraindicação anatômica em relação ao remodelamento do VD e das características da valva tricúspide. No relato em questão, o procedimento ocorreu de forma favorável, sem intercorrências e o paciente apresentou rápida recuperação, recebendo alta hospitalar em 24 horas, sem complicações.

Outro ponto importante nesse paciente foi a diminuição dos sintomas clínicos após o procedimento. Em um artigo ^8^ foi comentado que a diminuição do refluxo caval venoso parece melhorar a resposta à terapia diurética, mesmo sem levar a uma redução marcante do remodelamento inverso do VD e AD.

É importante relatar algumas limitações: o artigo discute a utilização de implantes simultâneos em apenas um paciente, mas levando em consideração a gravidade do quadro e o ineditismo do procedimento, é importante relatar os achados para auxiliar a comunidade médica na tomada de decisão em situações semelhantes. Outro fator é que os dispositivos bicavais apresentam limitações, como a dificuldade de dimensionamento e ancoragem e o risco aumentado de embolização. ^4^ No relato do paciente em questão nada disso ocorreu, talvez por ter sido implantado o sistema de válvula autoexpansível de nitinol, especificamente projetado para as veias cavas superior e inferior, minimizando os riscos. Outro ponto salientado ^8^ é que o sistema TricValve tem a vantagem de não ser diretamente exposto à energia cinética do jato da RT, minimizando o estresse mecânico aplicado na prótese.

## Conclusão

Este é o primeiro caso de sucesso reportado na literatura médica do implante concomitante de válvulas heterotópicas bicavais (TricValve) e oclusor de AAE (LAmbre). São necessários estudos prospectivos com maior número de pacientes e acompanhamento a longo prazo para estabelecimento dos riscos e benefícios de procedimentos afins em pacientes com características clínicas semelhantes e na evolução hemodinâmica e clínica ao longo do tempo.

Fonte: Hospital Vaz Monteiro (HVM – Lavras/MG)
