# Supplementary material for: Oclusão do Apêndice Atrial Esquerdo e Implante de Sistema Bicaval: Relato do Primeiro Procedimento Transcateter Simultâneo em Paciente Idoso Grave
Source: Arq Bras Cardiol. 2023 Dec 7;120(11):e20230454. [Article in Portuguese] doi: 10.36660/abc.20230454 (PMC10763697; doi:10.36660/abc.20230454)
Supplement: Translation

# Translation: Left Atrial Appendage Occlusion and Implantation of a Bicaval System: Report of a Simultaneous Transcatheter Procedure in a Critically Ill Elderly Patient

**Authors:** Marcos Cherem, Carlos Eduardo Bernini, Jamil Abdalla Saad, Dirceu Barbosa Dias, Marcio Sérgio Carvalho Silva, Ruthnea Aparecida Lazaro Muzzi

**Affiliations:** 1 Brazil Departamento de Cardiologia, Hemodinâmica e Cirurgia Cardiovascular, Hospital Vaz Monteiro, Lavras, MG – Brazil; 2 Brazil Departamento de Hemodinâmica, Hospital Felício Rocho, Belo Horizonte, MG – Brazil; 3 Brazil Universidade Federal de Lavras, Lavras, MG – Brazil

**Keywords:** Atrial Appendage, Venas Cavas, Cardiac Catheterization/methods, Atrial Fibrillation, Tricuspid Valve Insufficiency

Atrial fibrillation is a common arrhythmia that is associated with an increased risk of death, stroke, and peripheral embolism. ^1^ Closure of the left atrial appendage (LAA) as a prophylactic strategy against thromboembolic events in patients with atrial fibrillation using percutaneous occluders is a minimally invasive treatment option, especially in patients with contraindications to anticoagulation and open surgery. ^2^

The management of severe symptomatic tricuspid regurgitation continues to be a major challenge. ^3^ In this context, the use of transcatheter devices, for example, heterotopic caval valve implantation to relieve central venous congestion, has emerged as a strategy for indirect treatment of the systemic effects of severe torrential tricuspid regurgitation. ^4^

We report the first case of a double percutaneous thoracic intervention, conducted simultaneously, with the goal of LAA occlusion and the implantation of a TricValve system in an elderly patient with a severe cardiac condition.

## Case Report

We report the case of an 87-year-old male patient who presented with severe torrential tricuspid regurgitation with important hemodynamic repercussions and conditions of right heart failure, episodes of pulmonary thromboembolism, and deep vein thrombosis in the lower limbs, in follow-up at the hospital’s cardiology service since 1996.

The patient had severe comorbidities, including permanent atrial fibrillation in use of prophylactic enoxaparin and impossibility of safe oral anticoagulation due to chronic renal failure. Furthermore, he concomitantly presented stage 3 essential hypertension, left ventricular hypertrophy, hypercholesterolemia, and venous insufficiency of the lower limbs. Although he was using methyldopa, amiodarone, bisoprolol, enoxaparin, epoetin, dapagliflozin, furosemide, spironolactone, propatylnitrate, atorvastatin, and allopurinol, he progressively developed severe tricuspid insufficiency, right heart chamber dilation, and congestion in the venae cavae and right atrium, which were refractory to optimized clinical treatment, with progressive worsening of renal function. The transthoracic echocardiogram revealed severe and maladaptive concentric hypertrophy of the left ventricle, with systolic involvement of the right ventricle, important tricuspid insufficiency, important biatrial enlargement, and moderate pulmonary hypertension.

Owing to the unacceptable risk for open heart surgery, double percutaneous treatment was indicated, which consisted of closing the LAA and placing a heterotopic valve (TricValve®) in the cavae.

Transesophageal echocardiography was performed, showing LAA with bilobed “chicken wing” anatomy, without thrombi. The procedure was performed under sedation. Initially, 4 venous introducers were inserted as follows: a 5F and an 8F in the right femoral vein, a 5F in the left femoral vein, and an 8F in the right internal jugular vein. Subsequently, transseptal puncture was performed to access the left atrium, using a transseptal puncture needle. Afterwards, we performed intracardiac echocardiography (ICE) and LAA catheterization by angiography, verifying the previous measurements. We continued with the placement of the delivery sheath in the LAA (Seldinger technique). Subsequently, the LAmbre® 3236 device was implanted, with 24% oversizing for deeper implantation in the LAA, supported on the upper lobe. The device was released with the “umbrella” deep and the disc well-positioned in the orifice, without residual shunt (Figure 1: 1A, 2D-F).

Subsequently, the ICE catheter was repositioned in the right atrium to visualize the superior vena cava. A Lunderquist guidewire was introduced into the right subclavian vein, and a pigtail catheter was inserted through the introducer of the left femoral vein into the right pulmonary artery. With the aid of the ICE and pigtail catheter, the superior vena cava valve (TricValve® 29 mm) was positioned and released, ensuring that the device bulb was in the appropriate position, between the innominate vein and the venoatrial junction. Following release, ICE confirmed the absence of residual shunt (Figure 1: 1B and 2G).

Subsequently, the ICE catheter was repositioned in the right internal jugular vein introducer and advanced into the right atrium. With adequate visualization of the junction of the inferior vena cava and right atrium and the pigtail catheter in the right pulmonary artery, the inferior vena cava valve (TricValve 39 mm) was placed, ensuring adequate flow in the suprahepatic veins without periprosthetic leakage (Figures 1: 1C and 2H). Following valve release, the Lunderquist guidewire and introducers were removed. The patient was discharged from the hospital using the same medications as before, and his condition was stabilized, with no hospitalizations due to decompensation since that time.

## Discussion

This report describes a successful case of simultaneous implant placement in an elderly patient with severe heart disease and comorbidities that contraindicated conventional surgery due to an elevated risk of complications and death.

The patient reported had tricuspid regurgitation as the underlying disease, as well as permanent atrial fibrillation. To reduce the chances of an embolic event, we opted for LAA occlusion with the LAmbre device. Transcatheter LAA occlusion has become increasingly popular as an alternative to anticoagulation for prophylaxis of thromboembolic events in patients with atrial fibrillation. ^1 , 5^ In a study including 60 patients undergoing percutaneous LAA occlusion, ^6^ the possibility of periprosthetic leakage was discussed. This did not occur in the reported case, nor did thrombus formation or cerebrovascular events, thus demonstrating the efficacy and therapeutic safety of using occluders, especially in patients with atrial fibrillation and an elevated risk of stroke. However, it has been emphasized ^7^ that, as with all interventional procedures, the learning curve plays an essential role in percutaneous LAA occlusion, and it must be performed by experienced operators with excellent abilities, in collaboration with a cardiology team, as observed in this case report, composed of an experienced multidisciplinary group.

Due to right ventricular systolic dysfunction and severe tricuspid regurgitation, with systemic repercussions, we decided to implant the TricValve system. As described in another study, ^4^ the heterotopic bicaval system emerged as a possible transcatheter strategy for indirect treatment of the systemic effects of severe tricuspid regurgitation, with characteristics that greatly reduce the risk of implant embolization. It does not require general anesthesia, and there are no anatomical contraindications in relation to right ventricular remodeling and the characteristics of the tricuspid valve. In this case report, the procedure occurred favorably, without complications. The patient recovered quickly and was discharged from the hospital within 24 hours, without complications.

Another important point in this case was that the patient’s clinical symptoms reduced after the procedure. Another article ^8^ commented that the reduction in vena cava reflux appears to improve the response to diuretic therapy, even without leading to a pronounced reduction in right ventricular and right atrial reverse remodeling.

It is important to mention some limitations. This article discusses the use of simultaneous implants in a single patient. Nonetheless, taking into consideration the severity of the condition and the novelty of the procedure, it is important to report the findings in order to assist the medical community in decision-making in similar situations. Another factor is that bicaval devices have limitations, such as difficulty in sizing and anchoring and increased risk of embolization. ^4^ This did not happen in the reported case, perhaps due to the fact that we implanted a nitinol self-expandable valve system, which was specifically designed for the superior and inferior vena cava, thus minimizing the risks. Another point worth highlighting ^8^ is that the TricValve system has the advantage of not being directly exposed to the kinetic energy of the tricuspid regurgitation jet, minimizing the mechanical stress applied to the prosthesis.

## Conclusion

This is the first successful case reported in the medical literature of simultaneous implantation of heterotopic bicaval valves (TricValve) and an LAA occluder (LAmbre). Prospective studies with larger numbers of patients and long-term follow-up are necessary to establish the risks and benefits of related procedures in patients with similar clinical characteristics, as well as hemodynamic and clinical evolution over time.

**Figure 1:** – 1: Intracardiac echocardiographic images demonstrating A) LAA occluder, with the disc positioned; B) TricValve device in the SVC; C) TricValve device positioned in the IVC. 2: Fluoroscopic images demonstrating D) Contrast injection through the pigtail catheter in the LAA (with “chicken wing” morphology); E) Release of the device within the LAA; F) Release of the LAmbre device; G) Release of the first TricValve device in the SVC; H) Release of the second device in the IVC. IVC: inferior vena cava; LAA: left atrial appendage; SVC: superior vena cava. Source: Hospital Vaz Monteiro (HVM), Lavras, Minas Gerais, Brazil.
